# Association between the levels of gamma-glutamyl transpeptidase and the risk of stroke: systematic review and meta-analysis

**DOI:** 10.1055/s-0045-1809936

**Published:** 2025-07-15

**Authors:** Gustavo Adolfo Vásquez-Tirado, Stefany M. Nieto-Rivera, Claudia Vanessa Quispe-Castañeda, Edinson Dante Meregildo-Rodríguez, Leslie Jacqueline Liñán-Díaz, Wilson Marcial Guzmán-Aguilar

**Affiliations:** 1 Universidad Privada Antenor Orrego, Facultad de Medicina Humana, Trujillo, Perú.; 2 Hospital Regional Docente de Trujillo, Unidad de Cuidados Intensivos, Trujillo, Perú.; 3 Hospital Regional de Lambayeque, Lambayeque, Perú.

**Keywords:** Gamma-Glutamyltransferase, Stroke, Ischemic Stroke, Hemorrhagic Stroke, Meta-Analysis

## Abstract

**Background:**

Stroke is influenced by numerous factors, both modifiable and non-modifiable. Among these, gamma-glutamyl transpeptidase (GGT) serves as a prognostic biomarker in cardiovascular diseases and, within this context, in neurological conditions like stroke.

**Objective:**

To determine whether an association exists between GGT and both ischemic and hemorrhagic strokes.

**Methods:**

A systematic and comprehensive literature search was conducted across 5 databases, encompassing studies published from their inception to January 28, 2024, following a population, exposure, comparator, outcome, and study (PECOS) framework. Ten primary studies meeting the eligibility criteria were selected.

**Results:**

Our findings, based on a meta-analysis of the ten studies, indicate an increased risk of ischemic and hemorrhagic strokes in patients with elevated GGT levels, after excluding outliers. The analysis demonstrated a significant association, with a relative risk of 1.42 (95%CI: 1.01–1.99; I ^2^ = 19%) for hemorrhagic stroke and 1.22 (95%CI: 1.10–1.36; I ^2^ = 49%) for ischemic stroke.

**Conclusion:**

Our study reveals an elevated risk of stroke in patients with high GGT levels, demonstrating a 42% higher likelihood of hemorrhagic stroke and a 22% increased risk of ischemic stroke.

## INTRODUCTION

Stroke continues to be a significant worldwide health concern, and its impact is expected to grow in the future due to ongoing demographic shifts, such as population aging and health transitions in developing countries. 1 2

In Caucasian populations, roughly 80% of strokes are ischemic, while approximately 20% are hemorrhagic, typically caused by intracerebral or subarachnoid hemorrhages. 3

Globally, the burden of stroke has grown significantly between 1990 and 2019, with a 70% increase in incidence and an 85% rise in prevalence. Stroke-related deaths escalated by 43%, while disability-adjusted life years (DALYs) associated with stroke increased by 32%. 4 5 6

Emphasizing the identification of modifiable risk factors is crucial for the prevention and management of ischemic and hemorrhagic strokes. Among these factors, hypertension remains a key target for intervention. Additionally, smoking, alcohol consumption, and the use of anticoagulant medications significantly contribute to the risk of developing these types of strokes. 7 Nonetheless, the precise impact of risk factors such as hypertension and alcohol consumption on the incidence of cerebral hemorrhage remains uncertain. 8 9

Gamma-glutamyl transpeptidase (GGT) is frequently used in clinical practice as a biochemical indicator of liver damage; however, it lacks specificity in determining the underlying cause. 10 11 12 Evidence indicates that elevated GGT levels are linked to a higher risk of liver and cardiovascular disease as well as all-cause mortality. 13

Studies have reported that individuals with elevated GGT levels have a higher likelihood of developing stroke during follow-up compared with those with lower levels. In patients with ischemic stroke, GGT activity shows a stronger correlation with cardioembolic stroke, largely attributed to the presence of atrial fibrillation, and this relationship seems to be independent. 14 However, the connection between GGT activity and other negative stroke outcomes, including recurrence and combined cardiovascular events, has yet to be fully understood. 15

Although there are some prior systematic reviews and meta-analyses (SR-Ms) evaluating this association, most present the overall measure of association for both ischemic and hemorrhagic strokes. While these stroke types share similar risk factors, it is less appropriate to group them together, particularly given that GGT is more strongly associated with ischemic stroke risk than with hemorrhagic stroke. Furthermore, previous studies often demonstrate methodological limitations. Thus, the primary objective of the present systematic review and meta-analysis is to incorporate more recent evidence while addressing methodological issues to evaluate whether elevated GGT levels are associated with an increased risk of stroke.

## METHODS

The research protocol was initially developed and has been registered and approved in the International Prospective Register of Systematic Reviews (PROSPERO) (CRD42023469881). This secondary research was conducted following the recommendations in the Cochrane Handbook for Systematic Reviewss, 16 Preferred Reporting Items for Systematic Reviews and Meta-Analyses (PRISMA) guidelines, 17 and A Measurement Tool to Assess systematic Reviews (AMSTAR) 2 criteria. 18

### Search strategy

We conducted a systematic search following our protocol across databases including MEDLINE (via PubMed), EMBASE, Scopus, ScienceDirect, Web of Science, and the Cochrane Library. Our search strategy incorporated medical subject headings (MeSH), Boolean operators, and free-text keywords. The research question was framed using the population, exposure, comparator, and outcome (PECOS) structure as follows: population: adult patients; exposure: elevated GGT levels; comparator: normal or low GGT levels; outcome: ischemic or hemorrhagic stroke; study design: case control, cohort, and cross-sectional studies. Keywords were meticulously selected to capture relevant exposures ( *gamma-glutamyl transpeptidase* OR *glutamyl transpeptidase* ) and outcomes ( *stroke* OR *cerebrovascular accident* ). A comprehensive description of the search strategy employed for each database is available in the **Supplementary Material** – available at https://www.arquivosdeneuropsiquiatria.org/wp-content/uploads/2025/05/ANP-2024.0389-Supplementary-Material.docx ( **Supplementary Material Table S1** ).

**Figure 1:**
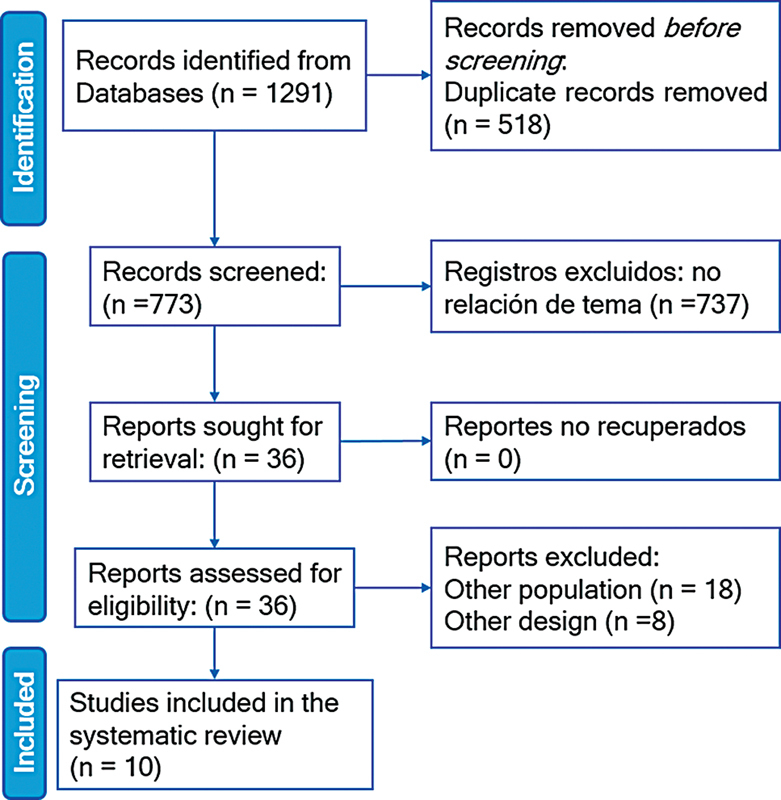
Preferred Reporting Items for Systematic Reviews and Meta-Analyses 2020 flow diagram of the selection process of the primary studies included.

A primary search was conducted in the aforementioned databases, followed by a secondary search within the identified records. The resulting studies were electronically organized using Zotero 6.0.15 (Corporation for Digital Scholarship), in which duplicate entries were removed. The remaining studies were then transferred to the Rayyan (Qatar Computing Research Institute) platform for further evaluation. At this stage, two authors (GVT and SNR) independently conducted a blinded review of titles and abstracts. Discrepancies were resolved through consensus, with a third author (EMR) acting as an arbitrator when necessary. Articles deemed eligible underwent a comprehensive complete review to confirm their inclusion. Additionally, reference lists and citations of the selected studies were manually reviewed to identify further relevant research. The entire selection process is illustrated in Figure 1 for better clarity.

### Eligibility criteria

Observational studies, including cohort, case-control, and cross-sectional designs, that examined the link between increased GGT levels and the likelihood of ischemic or hemorrhagic stroke as the primary outcome, were selected. The analysis considered studies involving adults aged 18 years or older. Studies published up to January 28, 2024, were reviewed without imposing restrictions on language or publication date. Studies such as case reports or series, duplicates, or those not meeting the inclusion criteria were excluded.

### Outcome

The primary outcome of interest in each study was the occurrence of ischemic or hemorrhagic stroke among patients with elevated GGT levels.

### Data extraction

Once the selected studies were identified, data extraction was performed during the full-text review. GVT and SNR independently conducted a blinded review of the full texts, ensuring the accuracy of the extracted data through comparison to minimize errors. The data were collected following the PECOS framework, incorporating information on population characteristics (clinical and laboratory features of the study cohorts), exposure (GGT levels categorized by quartiles as defined in each study), and outcomes (association measures such as odds ratio [OR], relative risk [RR], and hazard ratio [HR] when event data for exposed and unexposed groups were unavailable, along with potential confounding variables).

For dichotomous variables, association measures, including OR, RR, and HR, were utilized to assess the relationship between GGT levels and in-hospital mortality. Given the low stroke prevalence in the studied populations, OR, RR, and HR were considered interchangeable as effect measures. This approach facilitated a consistent and robust synthesis of evidence by integrating findings across studies employing different metrics. 19

### Statistical analysis

The pooled relative risks (RRs) from all included studies were calculated using R version 4.2.2 (R Foundation for Statistical Computing). Adjusted RRs were combined through the inverse variance method for meta-analysis, with 95%CIs calculated. The results of the quantitative synthesis were visually represented using forest plots generated with the *metagen* library. 20 The Inverse Variance (IV) method with Restricted Maximum Likelihood (REML) estimation for tau 2 was applied. Heterogeneity among the studies was analyzed using Cochran's Q test and Higgins' I ^2^ statistic. A random-effects model with Hartung-Knapp (HK) adjustment was implemented.

### Sensitivity analysis

When substantial heterogeneity was detected (I ^2^ > 40%), a sensitivity analysis was conducted. This analysis involved Influence Analysis using the *InfluenceAnalysis* function and Graphic Display of Heterogeneity (GOSH) through the *gosh.diagnostics* function in R, version 4.2.2. 21

### Quality assessment

For this systematic review and meta-analysis, the risk of bias was assessed using the Risk of Bias in Non-Randomized Studies – of Exposures (ROBINS-E; The Cochrane Collaboration) tool, specifically designed for observational research. Additionally, a funnel plot was generated, and Egger's test was performed to evaluate the presence of publication bias. 22 If evidence of publication bias was identified, its potential impact on the meta-analysis results was assessed using the trim-and-fill method. 23 In cases in which publication bias was detected, the trim-and-fill method was considered as a corrective measure. Any disagreements among the reviewers were resolved through discussions with the lead researcher (EMR).

## RESULTS

### Study selection and characteristics

A total of 1,291 primary articles were assessed across the databases consulted, after which 518 duplicates were excluded. Of the remaining 773 articles, 737 were excluded after title and abstract screening, resulting in 36 articles selected for full-text review, 26 of which were excluded due to study designs or populations that did not align with the inclusion criteria. Ultimately, 10 studies were included in the systematic review 24 25 26 27 28 29 30 31 32 33 ( Figure 1 ).

The 10 analyzed studies cover various aspects related to stroke and GGT. These studies originate from several countries, including Korea, 25 South Korea, 30 31 32 Finland, 33 Japan, 28 Austria, 26 the United Kingdom, 27 and Germany, 29 besides a multicentric study. 24 The total number of participants across all studies is approximately 1,072,267. The studies investigate the association between GGT levels and the risk of stroke, mortality, and other cardiovascular events, with results indicating a link between elevated GGT levels and a heightened risk of both stroke and mortality. Some studies also highlight the importance of GGT variability and its relationship with cardiovascular event risk. Overall, the findings underscore the potential role of GGT as a marker for stroke risk and other cardiovascular diseases ( **Supplementary Material Table S2** from the **Supplementary Materials** ).

### Risk of bias assessment

The assessment was conducted using the ROBINS-E tool, 34 This tool assesses observational studies across seven domains to determine their risk of bias. In the current analysis, eight studies were identified as having a low risk of bias. 24 25 26 27 28 32 33 articles were found to have a low risk of bias, while two 30 31 articles were classified as having some concerns. ( **Supplementary Material Table S3** ).

### Effect of GGT on hemorrhagic stroke

**Figure 2:**
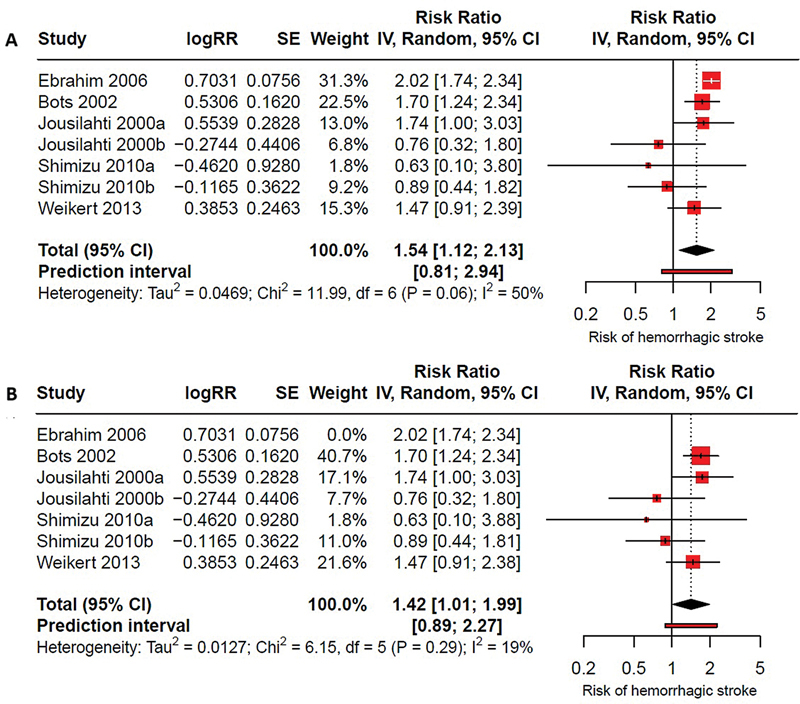
( **A** ) Forest plot of the association between high Gamma-glutamyl transpeptidase (GGT) levels and hemorrhagic stroke in adults. All studies were initially included in the meta-analysis, which revealed high heterogeneity. After identifying outliers using Graphic Display of Heterogeneity (GOSH) analysis, the study by Ebrahim (2016) 25 was excluded. ( **B** ) Forest plot following the exclusion of outliers, demonstrating the association between high GGT levels and hemorrhagic stroke with acceptable heterogeneity.

Of the studies identified, five provided data on GGT and hemorrhagic stroke. 24 25 28 29 33 However, Jousilahti et al. 33 and Shimizu et al. 28 considered two separate studies, as they report data for both men and women which were analyzed as independent primary studies. Elevated GGT levels were found to be associated with a 54% increased risk of hemorrhagic stroke (RR: 1.54; 95%CI: 1.12–2.13; I ^2^ = 50%) ( Figure 2A ), indicating substantial heterogeneity.

Through sensitivity analyses it was observed that Ebrahim et al. 25 behaved as an outlier. After excluding this study, the association persisted, showing a 42% increased risk with a significant reduction in heterogeneity (RR: 1.42; 95%CI: 1.01–1.99; I ^2^ = 19%) ( Figure 2B ).

### Effect of GGT on ischemic stroke

**Figure 3:**
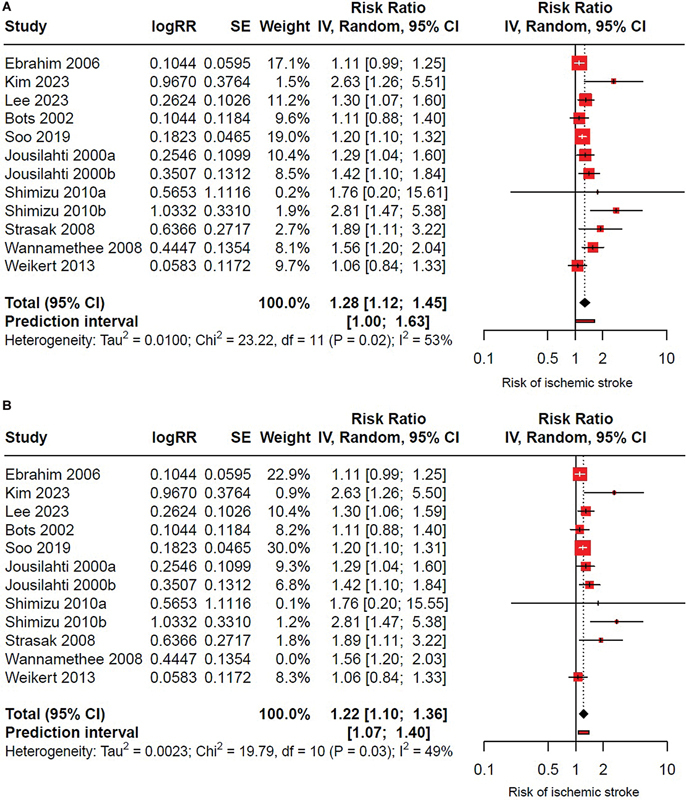
( **A** ) Forest plot of the association between high GGT levels and ischemic stroke in adults. All studies were initially included in the meta-analysis, which revealed high heterogeneity. After identifying outliers using GOSH analysis, the study by Wannamethee (2008) 27 was excluded. ( **B** ) Forest plot following the exclusion of outliers, demonstrating the association between high GGT levels and ischemic stroke with a slight reduction in heterogeneity.

A total of 10 studies were meta-analyzed, reporting data on GGT levels and the risk of ischemic stroke. 24 25 26 27 28 29 30 31 32 33 In two studies (Jousilahti et al. 33 and Shimizu et al. 28 ), data were provided separately for men and women; thus, these were treated as independent primary studies. Elevated GGT levels were related to a 28% increased risk of developing ischemic stroke (RR: 1.28; 95%CI: 1.12–1.45; I ^2^ = 53%), although with moderate heterogeneity ( Figure 3A ).

In the sensitivity analysis, after excluding studies identified as outliers (e.g., Wannamethee et al. 27 ), the risk remained consistent at 22% (RR: 1.22; 95%CI: 1.10–1.36; I ^2^ = 49%) with a slight reduction in heterogeneity ( Figure 3B ).

### Subgroup analysis

**Figure 4:**
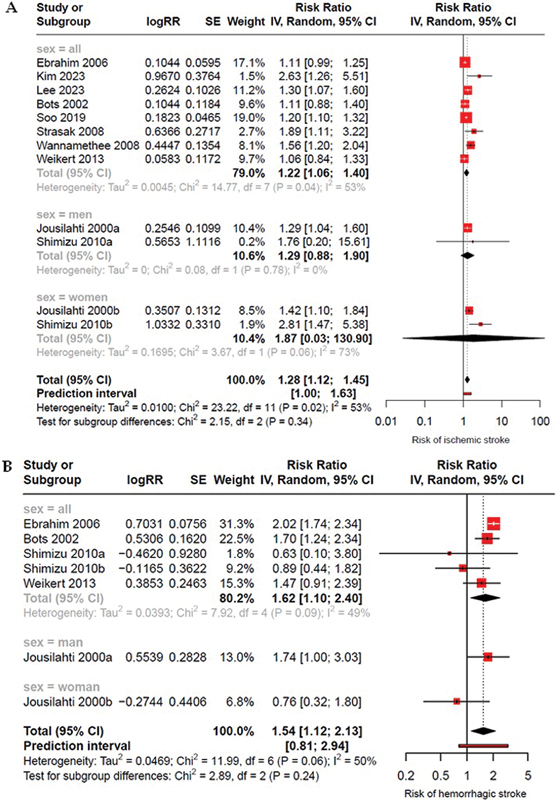
Forest plot of the association between high GGT levels and subgroup analysis by sex: ( **A** ) in ischemic stroke; ( **B** ) in hemorrhagic stroke.

Subgroup analyses stratified by sex revealed no significant association between elevated stroke risk, whether ischemic or hemorrhagic, and sex. The association with both ischemic and hemorrhagic stroke risk remained statistically significant in studies that included both men and women without stratifying results by sex (RR: 1.22; 95%CI: 1.06–1.40; I ^2^ = 53% for ischemic stroke, and RR: 1.62; 95%CI: 1.10–2.40; I ^2^ = 49% for hemorrhagic stroke). However, when the analysis was stratified by sex, the statistical significance was no longer observed ( Figure 4A,B ).

### Publication bias

**Figure 5:**
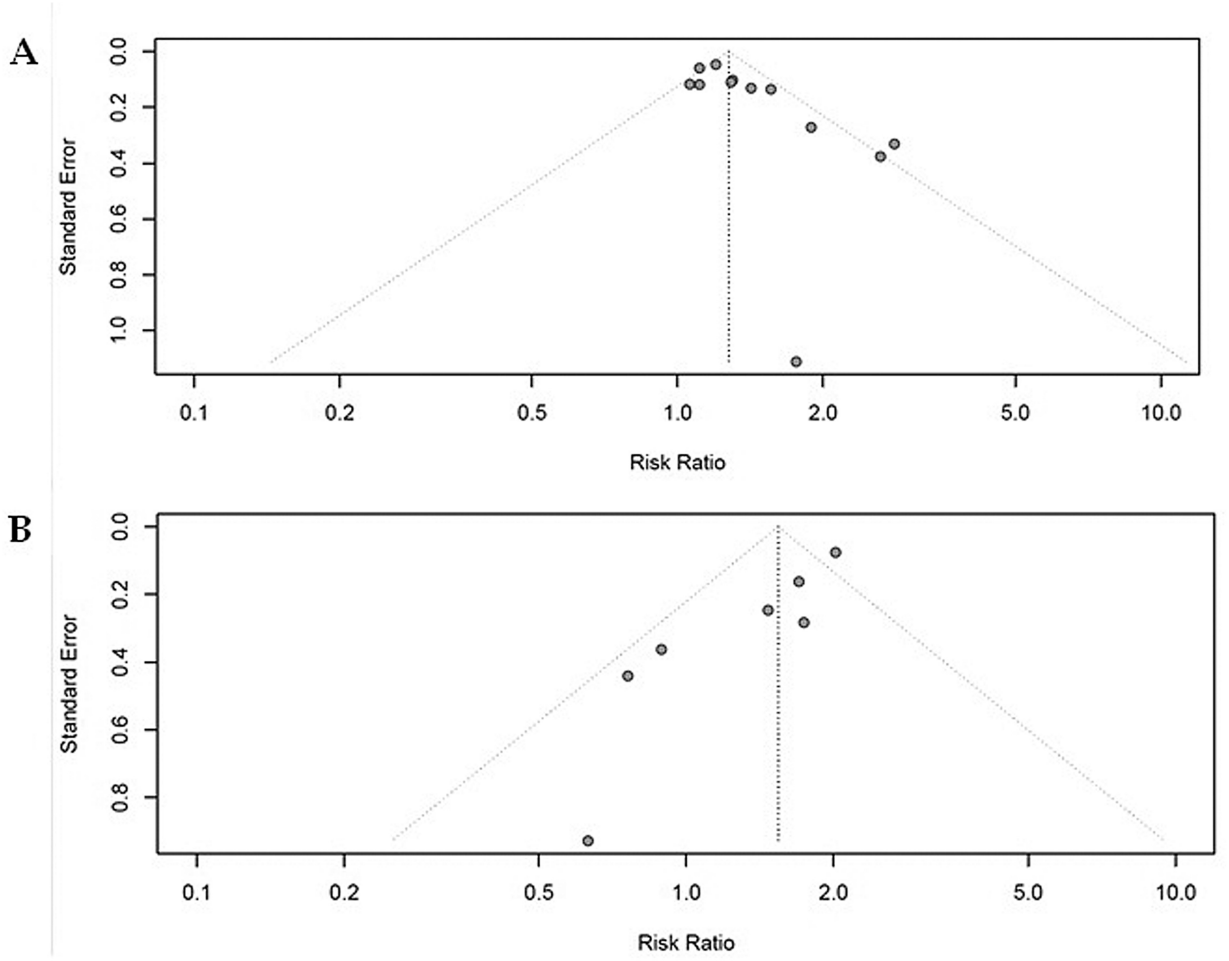
Funnel plot of the included studies in the meta-analysis on the association between high GGT levels and ( **A** ) ischemic stroke and ( **B** ) hemorrhagic stroke. Both plots suggest potential publication bias; however, this bias is not significant enough to impact the results.

In our analysis, visual evaluation of the funnel plot reveals a dispersion suggesting potential asymmetry, which was confirmed by Egger's test 22 for ischemic stroke (β₀: 1.72; 95%CI: 0.63–2.81; *p* < 0.05) and hemorrhagic stroke (β₀: -1.92; 95%CI: -2.67 to -1.17; *p* < 0.05), indicating possible publication bias ( Figures 5A,B ).

However, when assessing the impact of publication bias on the pooled RR in studies evaluating the association with both ischemic and hemorrhagic stroke using the trim-and-fill method (Duval and Tweedie 23 ), we found no significant impact ( **Supplementary Material Table 5S** ).

## DISCUSSION

Our SR-Ms demonstrate, after meta-analyzing 10 primary studies, a significant association between elevated GGT levels and an increased risk of both hemorrhagic stroke in 54% (RR: 1.54; 95%CI: 1.12–2.13; I ^2^ : 50%) and ischemic stroke in 28% (RR: 1.28; 95%CI: 1.12–1.45; I ^2^ : 53%) of the sample. These findings suggest that GGT may serve as a predictive biomarker for cardiovascular morbidity and mortality, rather than solely an indicator of liver damage or alcohol consumption, as traditionally considered.

These results align with those of prior studies suggesting a relationship between GGT levels and cardiovascular risk. For example, in a study by Zhang et al., 35 a meta-analysis was conducted to investigate the relationship between GGT levels and stroke risk, encompassing 5,707 stroke cases and 926,497 participants from 10 prospective studies. The findings revealed a significant positive association between elevated GGT levels and stroke risk (RR: 1.28; 95%CI: 1.16–1.43). Subgroup analyses demonstrated a consistent positive association across most groups, except for women (RR: 1.45; 95%CI: 0.90–2.34) and subgroups with a high number of stroke events (≥ 500) (RR: 1.25; 95%CI: 0.85–1.40). The study concluded that elevated GGT levels are significantly linked to an increased risk of stroke, independent of alcohol consumption, and highlighted potential gender and ethnic differences in this association.

Similarly, Rahmani et al. 36 performed a meta-analysis to explore the relationship between liver enzymes (GGT, alanine aminotransferase [ALT], aspartate aminotransferase [AST], and alkaline phosphatase [ALP]) and cardiovascular disease (CVD) mortality. The analysis included 23 studies involving 1,067,922 participants and identified a significant association between elevated GGT levels and increased CVD mortality risk (HR: 1.62; 95%CI: 1.47–1.7). No significant association was observed between ALT levels and CVD mortality (HR: 0.87; 95%CI: 0.73–1.07; *p* = 0.221; P-heterogeneity = 0.028). However, elevated baseline ALP levels and a higher AST/ALT ratio were directly linked to an increased risk of CVD mortality (HR: 1.45; 95%CI: 1.11–1.89 and HR: 2.20; 95%CI: 1.60–3.04), respectively. Conversely, no significant association was found between AST levels and CVD mortality risk (HR: 1.20; 95%CI: 0.83–1.73). A nonlinear association was observed between GGT and ALP levels and CVD mortality risk. The authors concluded that elevated GGT and ALP levels are directly associated with increased CVD mortality risk, reinforcing the role of liver enzymes as biomarkers for cardiovascular risk and associated mortality.

Fraser et al. 37 performed an SR-MA to evaluate the relationship between GGT levels and coronary and stroke events. The authors reported that a 1 U/L increase in GGT was associated with an increased risk of coronary heart disease (CHD) (RR: 1.20; 95%CI: 1.02–1.40), stroke (RR: 1.54; 95%CI: 1.20–2.00), and combined CHD or stroke (RR: 1.34; 95%CI: 1.22–1.48) in prospective studies. The meta-analysis included 10 prospective studies, and the heterogeneity decreased substantially when two studies involving Asian populations were excluded. Similar results were observed in non-drinker subgroups. Additionally, the analysis of ALT as a predictor of cardiovascular events yielded an HR of 1.18 (95%CI: 0.99–1.41) for CHD and 1.10 (95%CI: 0.89–1.36) for the combined outcome of CHD or stroke. The authors concluded that GGT is associated with vascular events independently of alcohol consumption, although the mechanisms underlying this relationship remain unclear and warrant further investigation.

The link between GGT and stroke may be attributed to its involvement in oxidative stress and inflammation, both of which are established risk factors for cardiovascular diseases. 38 GGT is involved in the metabolism of glutathione, a key antioxidant in defense against oxidative stress. Elevated GGT levels may reflect a pro-oxidant and pro-inflammatory state, contributing to the development and progression of atherosclerosis and ultimately increasing stroke risk. 31

Aspartate aminotransferase, also known as glutamate-oxaloacetate transaminase (GOT1), and alanine aminotransferase (ALT), also known as glutamate-pyruvate transaminase (GPT), along with GGT, are commonly recognized for their role in identifying liver injury. 39 Less well-known are the physiological functions of AST and ALT in regulating glutamate metabolism, wherein glutamate is converted to alpha-ketoglutarate and L-aspartate or L-alanine. Consequently, higher AST and ALT levels result in decreased blood glutamate levels, and vice versa. 29

The physiological role of GGT is to regulate antioxidant homeostasis by recycling extracellular glutathione (GSH). Gamma-glutamyl transpeptidase plays a crucial role in GSH and glutamate metabolism. 10 14 Elevated GGT levels have been identified as a risk factor for cardiovascular and all-cause mortality in population studies, independent of liver disease and alcohol intake. GSH, a well-known antioxidant, is a physiological reservoir of glutamate. Unlike elevated AST and ALT levels, which reduce blood glutamate levels, increased GGT levels stimulate glutamate synthesis. 40

Considering the association between GGT levels and stroke risk, measuring GGT levels could be useful as part of cardiovascular risk assessment in clinical practice. This approach may enable earlier preventive interventions in individuals with elevated GGT levels who do not exhibit symptoms of liver disease or excessive alcohol consumption.

### Strengths

Our study has several strengths. First, we implemented a comprehensive search strategy, encompassing six major databases, which allowed for a robust epidemiological evaluation of cause and effect. Second, we employed a rigorous methodology for conducting our review and meta-analysis, including a thorough quality assessment of the included studies and advanced statistical techniques to address heterogeneity. Third, our findings are robust and reliable, supported by meticulous heterogeneity assessments and the identification and exclusion of outliers, with consistent results across individual studies.

### Limitations

However, it is important to acknowledge some limitations in our study. First, not all primary studies used a similar GGT cutoff point to assess stroke risk; however, their evaluation by quartiles provides a close approximation in cases in which no specific cutoff was reported. Second, although sex is an important variable to assess, it could not be evaluated across all studies, as this distinction was not made in their respective primary designs. Third, not all studies included the optimal confounding variables in their original design.

In conclusion, our study suggests that elevated GGT levels are associated with an increased risk of stroke, raising the risk by 42% for hemorrhagic stroke and by 22% for ischemic stroke.
